# PI3Kδ is indispensable for CTL-mediated cytotoxicity

**DOI:** 10.1186/1471-2210-11-S2-A7

**Published:** 2011-09-05

**Authors:** Eva M Putz, Michaela Prchal, Olivia Simma, Florian Forster, Xaver Koenig, Roland Piekorz, Michael Freissmuth, Veronika Sexl, Eva-Maria Zebedin

**Affiliations:** 1 Institute of Pharmacology, Center of Physiology and Pharmacology, Medical University of Vienna, 1090 Vienna, Austria; 2 Institute of Pharmacology and Toxicology, University of Veterinary Medicine Vienna, 1210 Vienna, Austria; 3 Institute of Animal Breeding and Genetics, University of Veterinary Medicine, 1210 Vienna, Austria; 4 Institute of Hygiene and Applied Molecular Immunology, Medical University of Vienna, 1090 Vienna, Austria; 5 Institute of Biochemisty and Molecular Biology II, Heinrich Heine University, 40225 Düsseldorf, Germany

## Background

The expression of catalytic phosphoinositol-3-kinase isoform δ (PI3Kδ) is restricted to the haematopoetic compartment. Accordingly, PI3Kδ serves as a drug target to eliminate leukaemic cells. However, we previously showed that PI3Kδ is indispensable for the function of natural killer (NK)-cells [1]. Thus, the therapeutic success of PI3Kδ inhibitors is likely to be compromised by unintended side effects on the immune system. Besides NK-cells, CD8^+^ cytotoxic T-cells (CTLs) are well-known key players in natural host response against developing tumours and viral infections. In this study, we examine the role of PI3Kδ for CTL function and CTL-mediated tumour surveillance.

## Methods

PI3Kδ^−/−^ animals have been described in [2]. Flow cytometric lymphocyte characterization, the *in vivo* CTL-assay and MC-38 tumour model were done as outlined in [3]. Membrane capacitance and degranulation were measured by patch-clamp recordings and flow cytometry, respectively [1]; the mixed lymphocyte reaction was monitored as in [4]. *In vitro,* expanded CTLs were generated by stimulation with an anti-CD3ε antibody (0.5 µg/µL; BDPharmingen) and cultured for 3 days in T-cell medium containing 100 U/mL IL-2 prior to FACS analysis. For the *in vitro* cytotoxicity assay mice were immunized twice with the peptide SINFEKL. Peptide-reactive T-cells were generated by co-culturing splenocytes derived from immunized and control mice with SINFEKL-pulsed, irradiated splenocytes for 5 days. Peptide-reactive T-cells were co-cultured with CFSE-stained EL4 or EG7 cells in different effector:target-ratios. After 18 h peptide-specific killing was quantified via FACS.

## Results

Antigen-specific cytotoxicity of PI3Kδ^−/−^ CTLs was significantly reduced *in vivo* and *in vitro* as compared to wild-type. This defect translated into severely impaired CTL-mediated MC-38 tumour surveillance: tumours derived from PI3Kδ^−/−^ recipients were significantly bigger. PI3Kδ was required for full activation of CTLs and interfered with essential stages in the canonical killing pathway of CTL, e.g. with the endowment of their lytic machinery with key cytolytic molecules, with the production of interferon-γ and the fusion of lytic granules with the cellular membrane.

## Conclusions

Our findings are of particular interest for the clinical development, because specific inhibitors of PI3Kδ are entering clinical trials. Our observation shows that PI3Kδ is indispensable for CTL effector functions. Accordingly, long-term drug safety monitoring ought to include adequate measures to identify side effects resulting from impaired surveillance of viral infections and tumour cells.
